# Nutrition intervention is beneficial in oncology outpatients receiving radiotherapy to the gastrointestinal or head and neck area

**DOI:** 10.1038/sj.bjc.6601962

**Published:** 2004-06-29

**Authors:** E A Isenring, S Capra, J D Bauer

**Affiliations:** 1 School of Public Health, Queensland University of Technology, Brisbane, Australia; 2 Wesley Research Institute, Brisbane, Australia

**Keywords:** dietetics, nutrition support, radiation oncology

## Abstract

Malnutrition occurs frequently in patients with cancer of the gastrointestinal (GI) or head and neck area and can lead to negative outcomes. The aim of this study is to determine the impact of early and intensive nutrition intervention (NI) on body weight, body composition, nutritional status, global quality of life (QoL) and physical function compared to usual practice in oncology outpatients receiving radiotherapy to the GI or head and neck area. Outpatients commencing at least 20 fractions of radiotherapy to the GI or head and neck area were randomised to receive intensive, individualised nutrition counselling by a dietitian using a standard protocol and oral supplements if required, or the usual practice of the centre (general advice and nutrition booklet). Outcome parameters were measured at baseline and 4, 8 and 12 weeks after commencing radiotherapy using valid and reliable tools. A total of 60 patients (51M : 9F; mean age 61.9±14.0 years) were randomised to receive either NI (*n*=29) or usual care (UC) (*n*=31). The NI group had statistically smaller deteriorations in weight (*P*<0.001), nutritional status (*P*=0.020) and global QoL (*P*=0.009) compared with those receiving UC. Clinically, but not statistically significant differences in fat-free mass were observed between the groups (*P*=0.195). Early and intensive NI appears beneficial in terms of minimising weight loss, deterioration in nutritional status, global QoL and physical function in oncology outpatients receiving radiotherapy to the GI or head and neck area. Weight maintenance in this population leads to beneficial outcomes and suggests that this, rather than weight gain, may be a more appropriate aim of NI.

The incidence of malnutrition in patients with cancer ranges from 40 to 80% (Ollenschlager *et al*, 1991) and most frequently occurs in patients with cancer of the gastrointestinal (GI) or head and neck area (Lees, 1999). Malnutrition increases the risk of infections, treatment toxicity and health-care costs and decreases response to treatment, quality of life (QoL) and life expectancy (Nitenberg and Raynard, 2000).

Radiotherapy treatment can cause side effects that may limit oral intake and lead to weight loss. It has been suggested that adequate nutrition support during radiotherapy can decrease the impact of side effects, minimise weight loss, improve QoL and help patients to recover from the radiotherapy more quickly (Polisena, 2000, p. 70). There is little evidence based on clinical research to support this. Those studies that have investigated nutritional problems in oncology patients often draw attention to the links between nutritional status and outcomes. Many focus on biochemical and clinical issues and overlook the service delivery and more qualitative aspects of care such as QoL.

Many of the studies investigating nutrition support in the oncology setting have focused on the effect of enteral and/or parenteral nutrition on patient outcomes with mixed outcomes. The effects on outcomes were mixed. However, many had study design limitations including: no controlled allocation (Barber *et al*, 1998); inadequate nutrition support in terms of frequency of contact with the dietitian (Evans *et al*, 1987); small sample sizes (Wigmore *et al*, 1996); differing nutrition regimens and lack of standardisation of oral diets; and excluding those subjects who were lost due to attrition (Ovesen *et al*, 1993).

Traditionally, body weight and body mass index (BMI) have been used as outcome measures in dietetic practice, but these measures do not reflect the body composition changes that may occur during chronic disease such as cancer. It is the loss of fat-free mass (FFM) that is responsible for the reduced functional status, increased mortality and other negative outcomes associated with malnutrition (Tchekmedyian *et al*, 1992). Body fat is easier to gain than FFM, so studies that show improved body weight may not translate into reductions in morbidity or improvements in functional status.

The aim of this study was to determine the impact of early and intensive nutrition intervention (NI) on a range of outcomes including body weight, body composition, nutritional status, global QoL and physical function compared to usual practice in oncology outpatients receiving radiotherapy to the GI or head and neck area.

## SUBJECTS AND METHODS

Ethical approval was granted for this study from the Queensland University of Technology University Human Research Ethics Committee and The Wesley Hospital Multidisciplinary Ethics Committee and informed consent was obtained from all participants. A prospective, randomised-controlled trial was conducted. All outpatients commencing at least 20 fractions of radiotherapy to the GI or head and neck area at a private Australian radiation-oncology facility during a 12-month period were eligible for inclusion. Persons were deemed ineligible if they were: under the age of 18 years; hospital inpatients for greater than 5 days; receiving enteral or parenteral nutrition; or not able to provide informed consent.

In all, 78 consecutive patients were eligible for inclusion. Of these, 60 patients (51M : 9F; mean age 61.9 ±14.0 years) consented to the study and were randomised to receive either NI (*n*=29) or UC (*n*=31). A total of 88% of subjects were receiving radiotherapy to the head and neck (15% parotid, 13% oesophagus, 13% neck, 10% mouth, 8% vocal cords and 29% other head and neck areas) and 12% of patients were receiving radiotherapy to the abdominal or rectal area. In total, 47% of subjects were being treated with postoperative radiotherapy, 3% receiving preoperative radiotherapy and the remaining 50% received radiotherapy only and had no plans for surgery. Subject characteristics at baseline are presented in Table 1

**Table 1:** Baseline characteristics for subjects receiving NI and UC

**Variable (n)**	**NI (29)**	**UC (31)**
Gender (M : F)	24 : 5	27 : 4
Age (years)	60.6±15.6	63.3±12.5
Weight (kg)	74.8±7.8	77.6±18.2
Height (cm)	174.5±7.2	171.8±9.2
BMI (kg/m^2^)	25.2±4.4	26.4±4.5
PG-SGA score	7.1±6.1	5.9±4.3
SGA-A (well nourished)	17 (59)	22 (71)
B (suspected or moderately malnourished)	9 (31)	8 (26)
C (severely malnourished)	3 (10)	1 (3)
Percentage weight loss past 6 months	2.6 (0, 20.0)	3.6 (0, 12.6)
Global QoL score	67.7±18.8	75.3±19.2

NI=nutrition intervention; UC=usual care; BMI=body mass index; SGA=Subjective Global Assessment; PG-SGA=Patient-Generated-Subjective Global Assessment; QoL=quality of life; s.d.=standard deviation.

Continuous variables presented as mean±s.d. for normally distributed variables or median (range) for data that are not normally distributed. Categorical variables are presented as counts (%).

. According to Subjective Global Assessment (SGA), 65.0% (*n*=39) of subjects were well nourished and 35.0% (*n*=21) malnourished, of which 28.3% (*n*=17) were moderately nourished or suspected of being malnourished and 6.7% (*n*=4) were severely malnourished. Six subjects were lost to follow-up. There were no significant differences between the types of tumour and the fraction and dose of radiotherapy of subjects receiving NI or UC. There were no significant differences in baseline characteristics between subjects that were lost to follow-up and those that completed the study.

### Nutrition intervention

Patients received individualised NI in the form of regular and intensive nutrition counselling by a dietitian, following a predetermined standard nutrition protocol, the Medical Nutrition Therapy (Cancer/Radiation Oncology) protocol of the American Dietetic Association (ADA) (Gillbreath *et al*, 1998) for the 12-week study. This protocol included general guidelines referring to the time and frequency of dietitian consultations, data to be collected during the nutrition assessment and NI strategies, but did permit individualisation of the therapy to meet the specific needs of the patients. Nutrition counselling by the dietitian was provided within the first 4 days of commencing radiotherapy and weekly for the course of radiotherapy (approximately 6 weeks) and fortnightly for the remainder of the study period. Telephone reviews were conducted between nutrition counselling sessions. Individually tailored sample meal plans, recipe suggestions and hints to minimise the side effects of the tumour and therapy were provided. Standard patient handouts from the ADA Oncology Nutrition Dietetic Practice Group, as well as snack and high energy and protein exchange lists, were used. If deemed appropriate, the dietitian would provide a weekly supply of oral nutrition supplements for up to 3 months.

### Usual care

The UC group received the UC of that centre, that is, education by the nurses, provision of the resource ‘Understanding Nutrition – a booklet from the Queensland Cancer Fund’ and oral nutrition supplement samples. Compared to the NI group, those receiving UC received less nutrition assessment, no individualisation of nutrition advice and less follow-up. Patients receiving radiotherapy to the head and neck area were automatically referred to an outpatient dietitian. Those receiving radiotherapy to areas other than the head and neck could also request a referral to the outpatient dietitian. These patients received the UC of that service, which was a maximum of two dietetic consultations.

### Data collection

The following outcomes were measured: body weight and FFM (foot-to-foot bioelectrical impedance analysis (BIA)); nutritional status (scored Patient-Generated-Subjective Global Assessment (PG-SGA)); and global QoL (European Organisation for the Research and Treatment of Cancer (EORTC) QLQ-C30). Outcomes were assessed at the commencement of radiotherapy and 4, 8 and 12 weeks after commencing treatment.

*Foot-to-foot BIA*: is a recent development in BIA technology and is so named because an electric current is induced and the voltage drop measured via four metallic footplates situated on top of a conventional weighing scale. We have previously shown that foot-to-foot BIA is acceptable at the group level in measuring total body water and, hence FFM, in oncology patients receiving radiotherapy (Isenring *et al*, 2004).

*Scored PG-SGA*: has been developed for use in the cancer population (Ottery, 2000, p. 12) and is an adaptation of the validated nutrition assessment tool, SGA (Detsky *et al*, 1987). Patient-Generated-Subjective Global Assessment score, correlated with objective nutrition parameters (% weight loss, BMI), QoL, morbidity (survival, length of stay), has a high degree of inter-rater reproducibility and a high sensitivity and specificity when compared with other validated nutritional assessment tools (Bauer *et al*, 2002; Isenring *et al*, 2002). Each subject was classified as either well nourished (SGA A), moderately nourished or suspected of being malnourished (SGA B), or severely malnourished (SGA C), and in addition, a total PG-SGA score was calculated. For each component of the PG-SGA, points (0–4) are awarded depending on the impact on nutritional status. Typical scores range from 0 to 35 with a higher score reflecting a greater risk of malnutrition and scores ⩾9 indicating a critical need for NI and symptom management.

*EORTC QLQ-C30 (version 3)*: is a validated QoL assessment tool and was completed as described by the authors (Aaronson *et al*, 1993). This patient-based instrument is comprised of 30 items making up five functional scales (physical, role, cognitive, social, emotional), three symptom scales (fatigue, pain and nausea/vomiting), and global health status and global QoL scales. Physical function was also assessed using this tool. QLQ-C30 results are linearly converted to a score out of one hundred, with a higher score reflecting a higher QoL. Questionnaires were scored and transformed using the QLQ-C30 scoring manual (Fayers *et al*, 1999).

### Statistical analysis

All analyses were performed on an intention-to-treat basis. Repeated measure analyses were carried out for weight, nutritional status and global QoL using SPSS version 10, 2000 (SPSS Inc., Chicago, IL, USA). Weight gain or a weight loss of less than 1 kg over a 2- or 3-month period was classified as weight stable (Rosenbaum *et al*, 2000). The proportion of weight stable patients receiving either NI or UC was calculated using *χ*^2^ tests. More than 10% of the data were missing for FFM and so repeated measures using a different mathematical modelling approach was carried out using SUDAAN version 7.5.2A. This is a generalised estimating equations approach that permits the inclusion of subjects with incomplete data records. Hence, the analyses were based on 60 subjects who contributed between one and four time points of information. Statistical significance was reported at the conventional *P*<0.05 level (two-tailed).

## RESULTS

**Figure 1:**
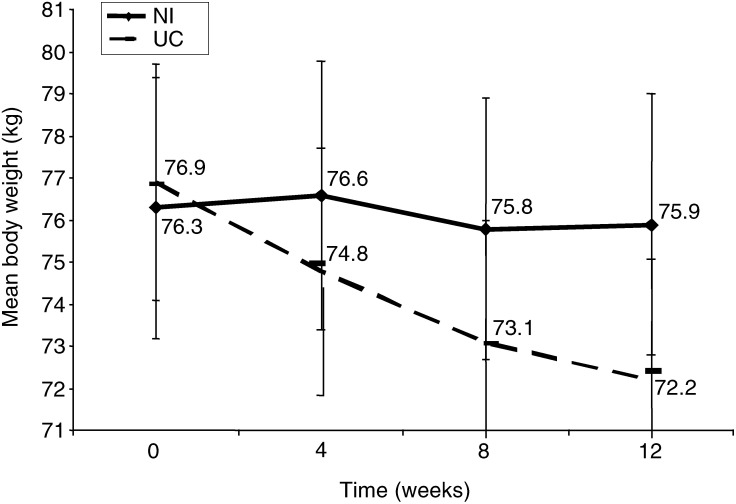
Mean body weight (s.e.m.) for ambulatory radiation-oncology patients receiving either NI or UC.

The NI group maintained body weight over 12 weeks (mean change=−0.4 kg) compared with those receiving UC who had a significantly greater deterioration in weight (mean change=−4.7 kg) (*P*<0.001) (Figure 1

). Significantly more patients in the NI group were weight stable compared with the UC group (Table 2

**Table 2:** Proportion of weight losing and weight stable subjects receiving either NI or UC

**Group**	**Weight stable**^a^	**Weight losing**^b^
NI	13 (24%)	12 (22%)
UC	6 (11%)	23 (43%)

NI=nutrition intervention; UC=usual care.

*P*-value=0.016 based on *χ*^2^ analyses.

a Weight stable=weight gain or weight loss <1 kg.

b Weight losing=weight loss of >1 kg.

). Changes in FFM over time were clinically significant with the NI group resulting in a mean gain of 0.5 kg and the UC group a mean loss of 1.4 kg FFM over 12 weeks, but this difference did not reach statistical significance (*P*=0.195).

**Figure 2:**
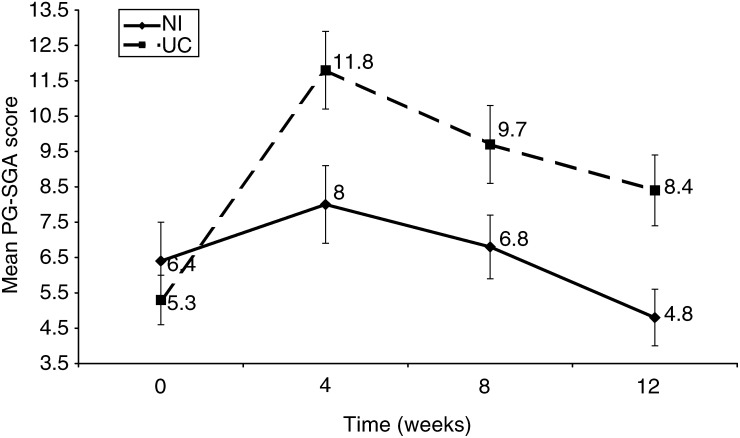
Mean (s.e.m.) PG-SGA score for ambulatory radiation-oncology patients either receiving NI or UC.

**Figure 3:**
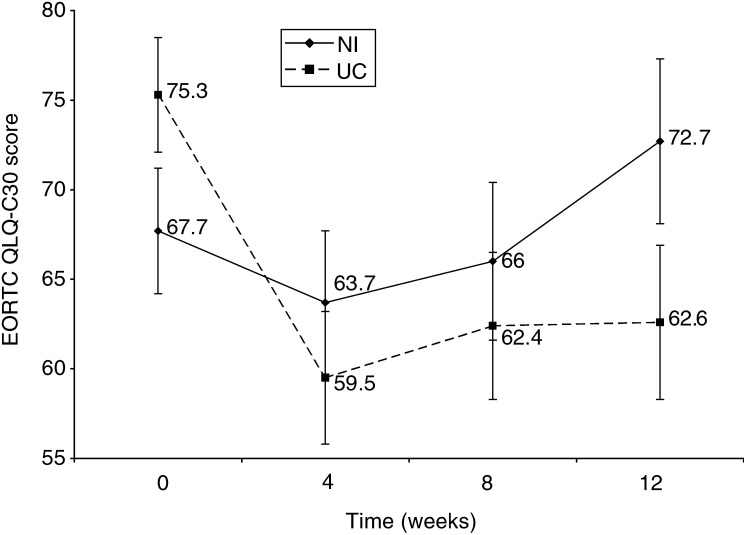
Mean (s.e.m.) EORTC QLQ-C30 score assessing global QoL for 54 ambulatory radiation-oncology patients receiving either nutrition NI or UC.

Those receiving NI had a significantly smaller deterioration in nutritional status as measured by PG-SGA score than those receiving UC (*P*=0.02) (Figure 2

). The NI group also had a significantly smaller decrease and faster recovery in global QoL (*P*=0.009) and in physical function (*P*=0.012) over time compared with the UC group (Figures 3

and 4

**Figure 4:**
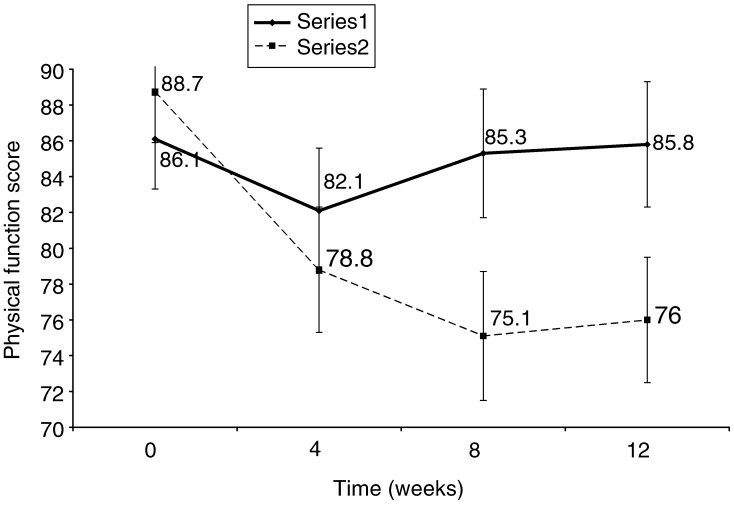
Mean (s.e.m.) physical function for 54 ambulatory radiation-oncology patients receiving either NI or UC.

).

## DISCUSSION

The aim of this study was to determine the impact of early and intensive NI on a range of outcomes including body weight, body composition, nutritional status, global QoL and functional status compared to usual practice in oncology outpatients receiving radiotherapy to the GI or head and neck areas.

### Body weight

Treatment-related side effects of patients receiving radiotherapy to the GI or head and neck area peak around two-thirds of the way during radiotherapy and continue for 2 or more weeks after completing treatment (Polisena and Wade, 1993). This is reflected in the current study with both NI and UC groups losing weight between the 4- and 8-week period. However, the NI group then regained weight. Providing intensive nutrition support with regular follow-up helped attenuate the natural weight loss history of treatment compared to those subjects in the UC group who reflected the typical decreases in body weight (Figure 1). This is confirmed by the data in Table 2, which showed that more weight stable subjects were receiving NI and more of the weight losing subjects were receiving UC.

The results of the current study are important because it is one of the first randomised-controlled trials that demonstrate beneficial outcomes in those receiving NI and contrasts with the conclusions of other nutrition support trials. In a review of 11 randomised-controlled trials in patients with cancer, it was concluded that oral nutrition supplements failed to improve weight, body composition or functional outcomes in patients with cancer (Stratton and Elia, 1999). Other studies have found that the best-case scenario was slowing the rate of weight loss in patients receiving chemotherapy despite an increase in protein and energy intake (Evans *et al*, 1987; Ovesen *et al*, 1993). Few studies have demonstrated the benefits of NI. A Cochrane review (Baldwin *et al*, 2004) investigating the impact of dietary counselling with and without oral nutrition supplements in malnourished patients concluded that nutrition supplements were more important than dietary counselling in maintaining body weight, but there were insufficient data to conclude whether the supplements decreased morbidity and mortality.

An important limitation of these studies was that there was insufficient description of what dietetic counselling involved: the frequency of contact and follow-up. Capra *et al* (2002) suggest that a failure to implement adequately the nutrition prescription and monitor compliance of this prescription could be responsible for the ‘negative’ results from dietary studies and highlights that these studies generally overlook patient-focused outcomes such as QoL and function status.

The suggested reasons as to why the current trial has been successful in maintaining body weight in the NI group compared with the UC group is the intensity and frequency of nutrition counselling. There was also a substantial follow-up period even after completing radiotherapy. The benefits appear to be due to the minimisation of eating difficulties typically experienced by patients receiving radiotherapy to the GI and head and neck area, rather than helping cachectic patients maintain weight. Treatment side effects, early satiety, fatigue and anorexia are possible to ameliorate with the appropriate dietary intake (Capra *et al*, 2002).

### Body composition

Generally, professional opinion considers changes of 0.5–1 kg in FFM to be clinically significant (Barber *et al*, 1999a, 1999b). Clinically significant differences in FFM were observed between the groups with a mean increase in FFM of 0.4 kg in the NI group *vs* the UC group, which experienced a mean decrease of 1.4 kg FFM. These values are similar to that found by May *et al* (2002) during a randomised-controlled trial investigating the impact of an amino-acid-enriched oral supplement in patients with stage IV solid tumours over 24 weeks and observed clinically significant differences in FFM between groups (1.60±0.94 *vs* 0.48±1.08 kg; *P*=0.20).

The majority of intervention trials that have shown beneficial influences on FFM have involved specialised nutrition or pharmacological products and have often targeted patients with cancer cachexia (Simons *et al*, 1998; May *et al*, 2002; Fearon *et al*, 2003). There is limited data on the impact of early and intensive nutrition support by way of dietetic counselling to increase protein and energy intake and target eating problems, on body composition.

### Nutritional status

The scored PG-SGA is a valid and reliable measure of nutritional status and allows tracking of changes in nutritional status over short periods of time, unlike broader nutritional measures, such as BMI (Bauer *et al*, 2002). Although the scored PG-SGA is widely used as a nutritional assessment tool in oncology patients and has been adopted by the American Dietetic Association as the standard protocol for use in patients with cancer, there is limited data available on the PG-SGA for comparison with this study because few studies have used the PG-SGA as an outcome measure. This study found that patients in the NI group had less deterioration in nutritional status as indicated by a lower PG-SGA score compared to the UC group.

### Global QoL

Medical care is no longer evaluated solely by traditional biomedical indicators (Niezgoda and Pater, 1993) and there is now a focus to have a broader concept of patient outcomes such as QoL (Cella and Cherin, 1988). The impact of nutrition on QoL has not been well documented; however, there are several studies that have observed poorer QoL outcomes in malnourished patients when compared with well-nourished patients (Larsson *et al*, 1995; Ohrn *et al*, 2001). QoL is an especially important outcome measure for treatments that are not predicted to impact on disease progression and survival (Sanders *et al*, 1998). This would include nutrition support interventions where perceived benefits are related to QoL and functional status rather than mortality.

For both groups global QoL was at its lowest at 4 weeks. It appears that global QoL is negatively influenced by the side effects that patients experience. Ravasco *et al* (2003) observed significant increases in QoL in patients who were receiving radiotherapy to the GI or head and neck area and individualised oral nutrition support despite an increase in symptoms. From the known history of radiation, it is reasonable to predict that global QoL would be at its lowest points at 4 and 8 weeks. However, improvements are already being seen in global QoL by the 8-week period, especially in those subjects receiving NI. This may be due to adaptation processes affecting QoL. Hagedoorn *et al* (2002) propose that some patients with cancer may experience a response shift that is defined as the change in the meaning of an individuals self-reported QoL, for example, different points in time may have different meanings. It may also be due to the fact that by 8 weeks, all subjects had completed radiotherapy treatment and would be back in their own homes. Even though experiencing some side effects, not having to visit the radiation-oncology centre and being in familiar surroundings may lead to improvements in QoL (Hagedoorn *et al*, 2002).

### Physical function

Change in physical function could also be responsible for a proportion of the change in global QoL. Figure 4 shows that there was a significant difference in physical function between the NI and UC groups over the 12-week study. However, while the physical function remained low in the UC group, the NI group was experiencing some recovery. It is reasonable to hypothesise that physical function would impact on QoL due to its relationship to activities of daily living (Hagedoorn *et al*, 2002).

The results of this study suggest that early and intensive nutrition support help minimise the reduction in global QoL and physical function that generally accompanies radiotherapy. NI also results in a faster improvement in global QoL and physical function. This is supported by trials by Jamieson *et al* (1997) and Tchekmedyian *et al* (2003, abstract), who observed a relationship between nutrition support, weight gain and improved QoL. However, there are several studies that have not found an increase in QoL with weight maintenance or gain (Saunders *et al*, 1991; Ovesen *et al*, 1993; Keele *et al*, 1997).

### Limitations

A potential limitation of this study is that there was no true control group and that those receiving UC still received an intervention, although this was less intensive compared with the NI. The higher than anticipated s.e.m. for changes in body weight reflects the heterogeneous nature of subjects’ nutritional status, which ranged from severely malnourished to obese. However, even with the large s.e.m. the differences in body weight over time between the groups were significant. The lack of long-term follow-up is a limitation of the study and it is recommended that future studies investigate longer-term morbidity and mortality data.

## CONCLUSIONS

Early and intensive NI provides beneficial outcomes in terms of minimising weight loss, deterioration in nutritional status, global QoL and physical function in ambulatory oncology patients receiving radiotherapy to the GI or head and neck area. Weight maintenance in this population leads to beneficial outcomes and suggests that this, rather than weight gain, may be a more appropriate aim of nutrition support during radiotherapy.

### Implications for practice

Patients at risk of malnutrition, such as those receiving radiotherapy to the GI or head and neck area, should receive regular and individualised nutrition support that continues postradiotherapy as required. Where staff levels are not sufficient to allow for this level of nutrition implementation, it is recommended that screening and triage systems be implemented to ensure that those clients most in need of care receive a level that demonstrates outcomes.
